# Repairing split ends: SIRT6, mono-ADP ribosylation and DNA repair

**DOI:** 10.18632/aging.100389

**Published:** 2011-09-22

**Authors:** Michael Van Meter, Zhiyong Mao, Vera Gorbunova, Andrei Seluanov

**Affiliations:** Department of Biology, University of Rochester, Rochester, NY 14627, USA

**Keywords:** Aging, cancer, SIRT6, DNA repair, PARP1

## Abstract

The sirtuin gene family comprises an evolutionarily ancient set of NAD+ dependent protein deacetylase and mono-ADP ribosyltransferase enzymes. Found in all domains of life, sirtuins regulate a diverse array of biological processes, including DNA repair, gene silencing, apoptosis and metabolism. Studies in multiple model organisms have indicated that sirtuins may also function to extend lifespan and attenuate age-related pathologies. To date, most of these studies have focused on the deacetylase activity of sirtuins, and relatively little is known about the other biochemical activity of sirtuins, mono-ADP ribosylation. We recently reported that the mammalian sirtuin, SIRT6, mono-ADP ribosylates PARP1 to promote DNA repair in response to oxidative stress. In this research perspective we review the role of SIRT6 in DNA repair and discuss the emerging implications for sirtuin directed mono-ADP ribosylation in aging and age-related diseases.

## INTRODUCTION

Sir2 enzymes, or sirtuins, are NAD^+^ dependent protein deacetylases and mono-ADP ribosyltransferases which regulate lifespan in *S. cerevisiae* [1], *C. elegans* [2] and *D. melanogaster* [3]. In each of these systems, overexpression or hyperactivation of Sir2 or its homologs extends lifespan. In yeast, this lifespan extension is achieved by promoting genomic stability [4], regulating gene expression [5, 6] and suppressing the formation of extra-ribosomal circles [1, 7]. In the roundworm *sir-2.1* promotes longevity by modulating *daf-16* (FOXO) signaling [2] and regulating the proteostasis stress response [8]; Sir2 extends lifespan in the fruit fly by coordinating a different stress response - the dietary restriction pathway [3]. Mammalian genomes encode seven Sir2 homologs (SIRT1-7); while it is unclear whether overexpression or hyperactivation of any of these sirtuins can extend lifespan, there is evidence that these genes can protect against several age-related pathologies, including neurodegeneration [9], hearing loss [10], diet induced obesity [11, 12] and cancer [13-15].

Of the mammalian sirtuins, SIRT6 recapitulates many of the biological functions of Sir2 and its homologs in fly and worm. At the molecular level, SIRT6 regulates the expression of a large number of stress-responsive and metabolism related genes [16-18], promotes genomic stability [19, 20] and stimulates base excision [19] and double strand break DNA repair [21-23]. Consistent with these molecular functions, SIRT6 knock-out mice develop a degenerative disorder that in many ways resembles premature aging [19]. These mice exhibit kyphosis, cachexia, greying of the fur, decreased bone mineral density, hypoglycemia, chronic inflammation and a severely shortened lifespan. This progeria is completely penetrant in multiple genetic backgrounds [24].

To date, most of the molecular functions of SIRT6 have been ascribed to the protein's deacetylase activity; SIRT6 is known to deacetylate H3K9 [20], H3K56 [25, 26] and CtIP [22]*in vivo*. Very little had been known, however, about the biological significance of the protein's mono-ADP ribosyltransferase activity. Recently we reported that SIRT6 mono-ADP ribosylates PARP1 to stimulate DNA repair in response to oxidative stress [23]. In this research perspective, we will review the role of SIRT6 in DNA repair and discuss the emerging implications for sirtuin directed mono-ADP ribosylation in the context of aging and age-related disease.

### SIRT6 and double strand break repair

Maintenance of genomic stability is a challenge faced by all organisms. The most grievous challenge to genomic stability comes in the form of DNA damage, and, in particular, lesions which cause double strand breaks (DSBs) [27]. Unrepaired, DSBs can lead to a host of adverse cellular phenotypes including irregular gene expression, permanent cell cycle arrest, cell death and malignant transformation. For this reason, eukaryotes have evolved two independent pathways for repairing this dangerous form of damage: homologous recombination (HR) [28] and non-homologous end joining (NHEJ) [29]. Efficient DSB repair is often a limiting factor for longevity, and mutations in the core DSB repair enzymes frequently result in a variety of disease states including premature-aging and a predisposition for cancer, underscoring the importance of DSB repair in the context of health and aging [30, 31].

The first evidence that SIRT6 may impact on DSB repair came from the observation of genomic instability in SIRT6 knock-out mice; cells from these mice exhibit a high incidence of chromosomal rearrangement and breakage as well as a hypersensitivity to γ-irradiation [19]. Two subsequent studies noted that knockdown of SIRT6 in human cells similarly sensitized the cells to chemically induced DSBs, and also observed that SIRT6 is recruited to break sites after DNA damage [21, 22]. The first of these studies suggested that SIRT6 functions in NHEJ by stabilizing the NHEJ haloenzyme, DNA-PK, at the site of DSBs, and the second study revealed that SIRT6 promotes HR by deacetylating, and thereby activating, the end resection protein, CtIP. These studies provided the first mechanistic insight into how SIRT6 functions in DSB repair.

Our group recently published a report that further clarifies the role of SIRT6 in DNA repair and underscores the dynamic role that SIRT6 plays in promoting genomic stability, especially in response to oxidative stress [23]. Whereas previous studies had depleted SIRT6 to assess its role in DNA repair, we noted that overexpression of SIRT6 stimulated DSB repair through both the HR and NHEJ pathways by approximately 3-fold. Strikingly, when cells were pretreated with oxidative stressors prior to overexpression of SIRT6, DSB repair efficiency was massively stimulated by up to 16-fold, suggesting that SIRT6 specifically integrates stress signaling to prime the DNA repair machinery in response to oxidative stress. Consistent with this hypothesis, we observed that while SIRT6 is normally recruited to DSB sites relatively late, approximately 8 hours after induction of the DSB, pretreatment with paraquat resulted in an early wave of SIRT6 recruitment to the breakage points, within 30 minutes of inducing the break.

To assess which biochemical activity of SIRT6 mediated this stress response, we synthesized SIRT6 mutants which could catalyze only deacetylation or only mono-ADP ribosylation reactions, thereby uncoupling these two enzymatic activities. Intriguingly, both of these activities were required to stimulate DSB repair, indicating that SIRT6 played a role in DSB repair beyond interacting with DNA-PKcs and deacetylating CtIP. Consistent with this hypothesis, we found that SIRT6 mono-ADP ribosylates the upstream DSB repair factor, PARP1, at lysine 521, thereby stimulating its poly-ADP ribosylation activity. Importantly, this modification was required for SIRT6-mediated stimulation of DSB repair. Mechanistically, PARP1 enables several crucial interactions in the early stages of DSB repair by poly-ADP ribosylating itself and other protein substrates. Notably, PARP1 facilitates the recruitment of the MRN complex to DSBs [32], plays a role in the activation of ATM [33] and helps to direct the choice between the NHEJ and HR repair pathways. Additionally, PARP1 is required to promote a non-canonical form of NHEJ (Alt-NHEJ) [34]; consistent with this, SIRT6 can stimulate NHEJ in the absence of DNA-PK, an essential canonical-NHEJ enzyme, suggesting that SIRT6 can stimulate NHEJ through the alternative pathway.

**Figure 1:**
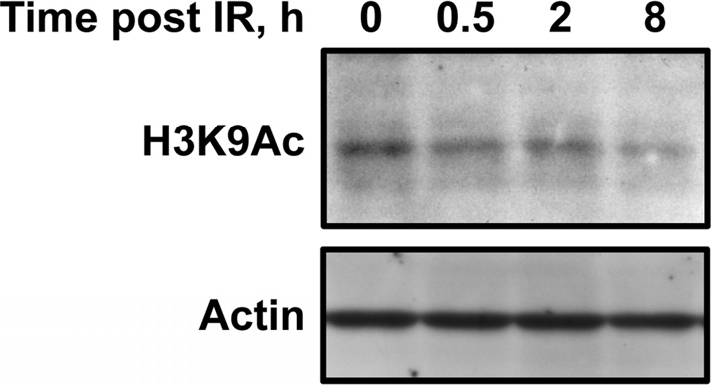
The second wave of SIRT6 recruitment to DSBs is concomitant with global deacetylation of H3K9 Human fibroblasts were exposed to γ-irradiation. Immunoblotting revealed that H3K9-Ac levels are reduced 8 hours following irradiation, concurrent with a second wave of SIRT6 recruitment to DSBs.

The interaction with PARP1 explains the early recruitment of SIRT6 to sites of DSBs under stressed conditions. Intriguingly, we also observed that H3K9 is globally deacetylated at a late, 8 hour post-break, time point (Figure 1). Previous studies have indicated that this deacetylation is dependent on SIRT6 [21]. Whereas the early interaction between SIRT6 and PARP1 likely functions to stimulate the repair process, it is possible that this later wave of histone deacetylation functions to restore the original chromatin structure of the locus or to condense chromatin to prevent further damage. Another intriguing possibility is that SIRT6 relocalization in response to DNA damage may affect gene expression. Consistent with this possibility, another mammalian sirtuin, SIRT1, relocalizes to DSB sites in response to damage; this relocalization is concomitant with deregulation of SIRT1-regulated genes and may contribute to several aging phenotypes [35]. It will be interesting to see if changes in gene expression are associated with SIRT6 relocalization in response to DNA damage, and what effect these changes may have on the cell.

The finding that SIRT6 primes the DSB repair machinery in response to stress suggests an energetically pragmatic paradigm, wherein certain DNA repair enzymes exist in a basal state under normal conditions, but can become activated in response to oxidative stress. Such a system would allow cells to stimulate DNA repair under conditions in which they are most likely to sustain DNA damage. This might be beneficial because it would allow the cell to conserve energy and curtail deleterious side-effects associated with chronic activation of DSB repair enzymes which can include oncogenic hyper-recombination and cell death. Constitutive activation of PARP1, for example, promotes cell death [36, 37]; SIRT6 mediated activation of PARP1 specifically in response to stress may represent a mechanism for controlling this response.

This adaptive response, wherein SIRT6 is mobilized following mild doses of oxidative stress to activate DNA repair machinery, in many ways resembles a hormetic response. Hormesis predicts that exposure to low levels of stress can result in favorable biological outcomes such as increased resistance to stress or extended lifespan; caloric restriction is a well characterized example of this phenomenon [38, 39]. At the cellular level, several studies have indicated that low levels of oxidative stress are beneficial to the cell [40, 41]. It is possible that SIRT6 mediates this hormetic response, providing a link between stress sensing pathways and/or NAD^+^ levels and DNA repair machinery. This raises the intriguing possibility of using pharmacological activators to stimulate SIRT6 activity as a means of slowing and attenuating the onset of age-related pathologies.

**Figure 2:**
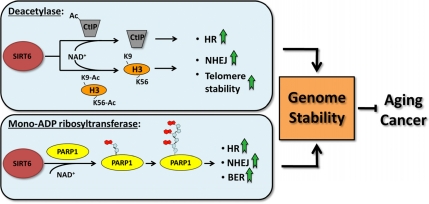
SIRT6 regulates genomic stability SIRT6 promotes genome stability by regulating DNA single-strand and double strand break repair pathways and by facilitating telomere maintenance. The deacetylase and the mono-ADP ribosyltransferase activities of SIRT6 both contribute to this function.

While SIRT6 has emerged as an important mediator of genome stability (Figure 2), there are still several questions with regards to the role that SIRT6 plays in DNA repair. In response to oxidative stress, SIRT6 is recruited to chromatin and mono-ADP-ribosylates PARP1, but what triggers this reaction? ATM, NF-κB and the MAPKs are stress responsive kinases which have been implicated in DSB repair or shown to interact with other sirtuins [42-44] - it is possible one or more of these proteins transduces stress signaling to SIRT6. Another intriguing question involves base excision repair (BER) - SIRT6^-/-^ mice exhibit a defect in BER, although the etiology of this deficiency remains unclear [45]. In response to single strand breaks, PARP1 binds to DNA and facilitates the recruitment of BER factors to instigate repair [46]. Could SIRT6 also promote BER through PARP1, and if so, would this response also be heightened in response to oxidative stress? Answering these and other related questions will provide a clearer understanding of the important role that SIRT6 plays in genome maintenance.

### Mono-ADP ribosylation

Although sirtuins are best understood in the context of protein deacetylation, the founding member of this gene family, Sir2, was first described as a mono-ADP ribosyltransferase [47]. This form of post-translational modification was first identified as a feature of several bacterial toxins, including diphtheria, cholera and pertussis. Subsequent studies revealed the presence of eukaryotic mono-ADP ribosyltransferases which function in multiple biological pathways, including signal transduction, gene expression and cellular differentiation [48, 49]. These transferases can broadly be sorted into distinct categories on the basis of homology and localization and include: classical mono-ADP ribosyltransferases (ARTs), certain members of the PARP family and sirtuins [48, 50].

The role of sirtuins as protein deacetylases has been well characterized. Briefly, sirtuins couple NAD^+^ hydrolysis with lysine deacetylation to generate deacetylated protein, and the metabolites O-acetyl-ADP-ribose and nicotinamide [51]. Sirtuin mediated deacetylation regulates a large array of biological processes. Many sirtuins, however, also possess a less characterized enzymatic activity, mono-ADP ribosylation [52]. In this context, sirtuins transfer the ADP-ribose moiety of NAD^+^ to acceptor proteins. Ribosylation activities have been reported for sirtuins in a wide range of organisms. For example the protozoan *T. brucei* Sir2 homolog mono-ADP ribosylates H2A and H2B in response to DNA damage [53]; yeast Sir2 catalyzes the transfer of ADP ribose to itself and histones [47]; and the mammalian sirtuin, SIRT4 ribosylates glutamate dehydrogenase to suppress insulin signaling in pancreatic β-cells [54]. Several studies have also suggested that many other sirtuins, including *E. coli* CobB, as well as mammalian SIRT1 and SIRT3, possess mono-ADP ribosyltransferase activity, although the biological significance of this activity remains unclear because no *in vivo* substrates have been identified for this reaction [52, 55]. Akin to Sir2, SIRT6 was first reported as an auto mono-ADP ribosyltransferase [56], and only later discovered to possess protein deacetylase activity [20].

Several recent reports have provided insight into the biochemistry of SIRT6-mediated mono-ADP ribosylation and have suggested that there are additional, as yet uncharacterized, substrates for this reaction. Crystallography of SIRT6 revealed several unique features, including the absence of a helix bundle that typically connects the Rossmann and zinc binding domains in other sirtuins [57]. This distinct structure favors the binding of NAD^+^ even in the absence of acetylated substrate, and as a result may facilitate mono-ADP ribosyltransfer reactions. Consistent with the notion that SIRT6 is well suited to catalyze mono-ADP ribosylation we recently reported that overexpression of SIRT6 selectively induces massive apoptosis in cancer cells but not non-cancerous cells and that this cytotoxicity is dependent on the mono-ADP ribosylation activity of SIRT6 [15]. As yet it is unclear exactly how SIRT6 mono-ADP ribosylation promotes death in cancer cells, but it appears to be independent of PARP1, suggesting that there are additional targets for SIRT6-mediated mono-ADP ribosylation *in vivo*.

In consideration of the dual enzymatic activities of sirtuins, early models predicted that the deacetylase activity of sirtuins may function to regulate gene expression, whereas the mono-ADP ribosylation activity may mediate DNA repair [58]. While the discovery of non-histone substrates for deacetylation, and the observation that SIRT4 ribosylates a metabolic enzyme suggests that this model may be simplistic, there is evidence that mono-ADP ribosylation is an important feature of DNA repair. Multiple studies have observed that mono-ADP ribose is transferred to histones in response to DNA damage [59-61], although it is unclear if this response is entirely mediated by sirtuins. The protozoan Sir2 homolog, TbSIR2RP1 ribosylates histones in response to DNA damage [53], and we recently demonstrated that SIRT6 mono-ADP ribosylates PARP1 to promote DNA repair in response to oxidative stress. Future studies will be required to reveal the full importance of sirtuin-mediated mono-ADP ribosylation reactions.

### Concluding remarks and prospectus

It is becoming clear that SIRT6 functions in multiple pathways related to aging by facilitating DNA repair, promoting telomere stability, attenuating NF-κB activity and regulating metabolism. Intriguingly, destabilization of any of these pathways can lead to the accumulation of aging related phenotypes [62-65]. It will be interesting to see whether SIRT6 overexpression or hyperactivity can ameliorate or delay the onset of age-related pathologies, possibly by stimulating hormetic response pathways. In this context we have shown that SIRT6 overexpression can improve the efficiency of NHEJ and HR, perhaps by mimicking an endogenous response to oxidative stress. In a separate study, we observed that SIRT6 overexpression induces massive apoptosis in cancer cells, but not non-cancerous cells. Finally, another group has indicated that SIRT6 overexpression protects against diet induced obesity in mice [12]. Collectively, these studies provide support for the hypothesis that SIRT6 may protect against aging or age-associated pathologies, although more evidence will be required to confirm this. It is worth noting that several studies have indicated that it may be possible to modulate SIRT6 activity using physiological or pharmacological interventions [66, 67]. It will be interesting to further assess whether modulating SIRT6 levels and activity can yield desirable clinical outcomes.
